# A Rare Presentation of Inflammatory Myofibroblastic Tumor in the Nasolabial Fold

**DOI:** 10.1155/2019/3257697

**Published:** 2019-01-23

**Authors:** Hind K. Alshammari, Haya F. Alzamami, Mona Ashoor, Wasan F. Almarzouq, Haitham Kussaibi

**Affiliations:** ^1^College of Medicine, Imam Abdulrahman Bin Faisal University, Dammam, P.O Box 66609, Saudi Arabia; ^2^Assistant Professor at Otolaryngology-Head and Neck Surgery, Imam Abdulrahman Bin Faisal University, Dammam, P.O Box 66609, Saudi Arabia; ^3^Assistant Professor at Pathology Department, Imam Abdulrahman Bin Faisal University, Dammam, P.O Box 66609, Saudi Arabia

## Abstract

Inflammatory myofibroblastic tumor (IMT) is a benign lesion that occurs most frequently in the soft tissues and viscera. In the head and neck region, the tumor has been reported to occur in the orbit, tongue, nasopharynx, larynx, and paranasal sinuses and the central nervous system. Despite being a benign lesion, it exhibits infiltrative and destructive behaviours, making histopathological examination necessary to confirm the diagnosis. We report the case of a 38-year-old female presented with a right nasolabial fold mass, which was confirmed histologically to be an IMT. Surgical excision of the mass was achieved through a sublabial approach with an uneventful postoperative period. To the best of our knowledge, this is the first reported case of an IMT in the nasolabial fold.

## 1. Introduction

Inflammatory myofibroblastic tumor (IMT) is a benign tumor-like lesion that histologically displays a wide range of spindle-shaped myofibroblastic proliferation accompanied by various amounts of chronic inflammatory infiltrates [1]. The lesion has been given multiple labels depending on the predominant cells seen in the histopathology of the tumor; this includes inflammatory pseudotumor, plasma cell granuloma, and lately, inflammatory myofibroblastic tumor [2]. This rare pseudotumor can often be misdiagnosed in the early stages of diagnosis, and it can resemble malignant tumors both clinically and radiologically [3]. We report a case of a 38-year-old female who presented with a right nasolabial fold mass, which was later confirmed to be an IMT. To the best of our knowledge, this is the first reported case of an IMT in the nasolabial fold.

## 2. Case Report

**Figure 1:**
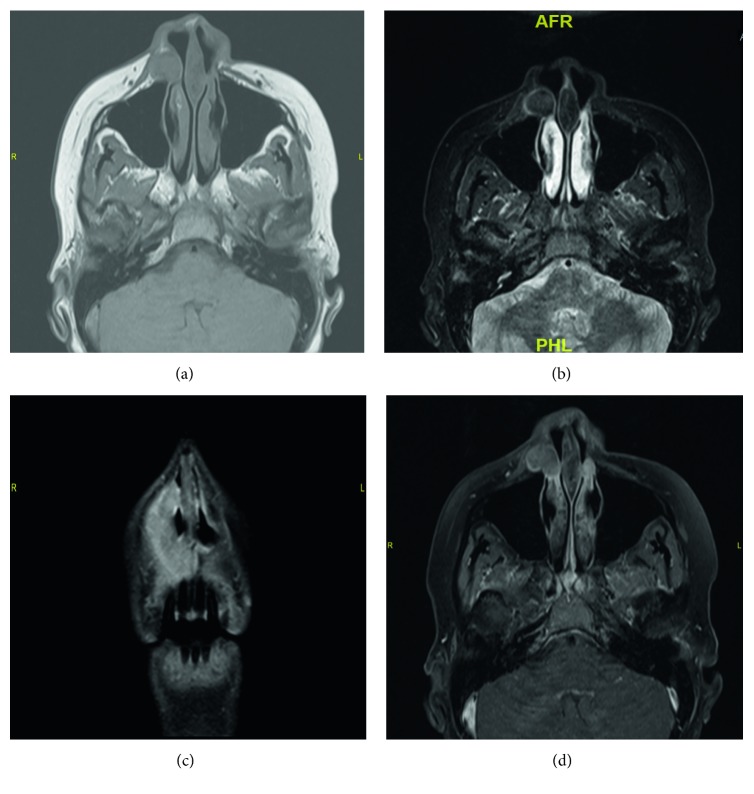
Magnetic resonance imaging. (a) Axial section shows well-defined subcutaneous soft tissue density located in the right nasolabial fold appearing isointensely on T1. (b) Hypointensity on T2. (c, d) Homogeneous enhancement after contrast.

A 38-year-old Saudi female presented in July 2014 complaining of bilateral nasal obstruction for 10 years, for which she sought medical attention and underwent septoplasty and functional endoscopic sinus surgery 7 years ago at a different institute. Postoperatively, the patient noticed minimal improvement in her symptoms with persistence of right nasal obstruction. She also noticed right nasolabial fold fullness for a year, which increased in size over time associated with right facial pain. Clinical examination revealed a swelling in the right nasolabial fold measuring 2 × 1 cm. It was tender to palpation, hard in consistency, but with normal overlying skin. Anterior rhinoscopic examination of the right nasal cavity revealed lateral nasal wall swelling obliterating 90% of the nasal vestibule with normal overlying mucosa and skin colour externally. Computed tomography (CT) and magnetic resonance imaging (MRI) revealed a well-defined subcutaneous soft tissue density measuring 1.6 × 1.6 cm located in the right nasolabial fold. The lesion demonstrated isointensity with no drop in fat saturation T1 but showed hypointensity on T2 and homogeneous enhancement postcontrast (Figure 1).

**Figure 2:**
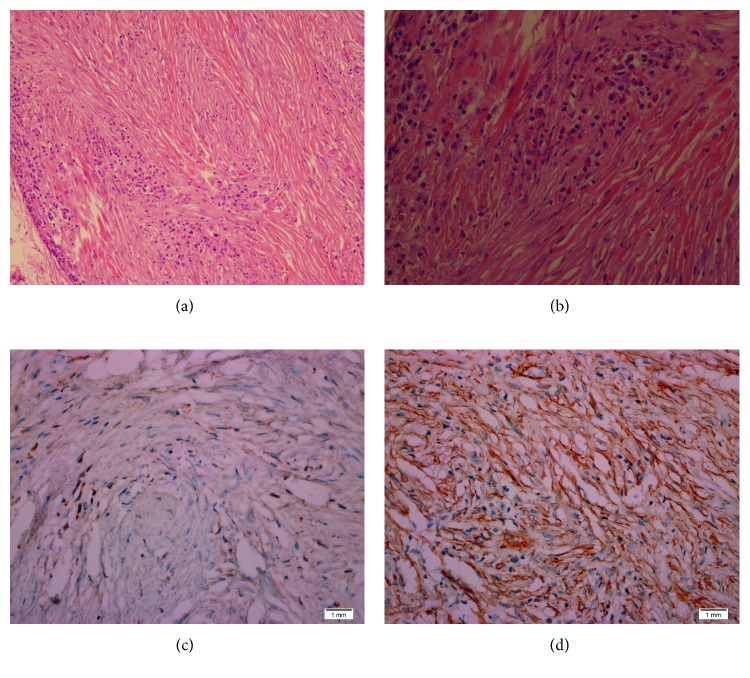
Histopathological examination of the mass revealed (a) 100x microscopic image showing spindle cells proliferation, forming fascicles and whorls on a background of collagen fibres, and (b) 200x microscopic image showing mixed inflammatory cells infiltration in the background including plasma cells and eosinophils. (c) Spindle cells are focally positive for anaplastic lymphoma kinase (ALK) by immunohistochemistry. (d) Spindle cells are diffusely positive for smooth muscle actin (SMA) by immunohistochemistry.

In August 2014, the patient underwent surgical excision of the mass through a sublabial approach, and the mass was found to be encapsulated with no infiltration to surrounding tissues and was excised completely with its capsule with uncomplicated postoperative period. Histopathological examination of the mass revealed spindle cells proliferation, forming fascicles and whorls on a background of collagen fibres. The fascicles were associated with foci of mixed inflammatory cells infiltrate composed mainly of lymphocytes and plasma cells, along with scattered eosinophils and neutrophils. The lesion infiltrates the surrounding striated muscles and fatty tissue with no infiltration to cutaneous and subcutaneous tissue. Moreover, immunohistochemical studies on the spindle cells revealed they are diffusely positive for vimentin and smooth muscle actin (SMA) (Figure 2) and focally positive for anaplastic lymphoma kinase (ALK). In contrast, they were negative for S100, CD34, P-catenin, CD99, and epithelial membrane antigen (EMA). The patient has been on regular follow-up visits at our clinic, and is now four years with no complaints or tumor recurrence.

## 3. Discussion

Inflammatory myofibroblastic tumors (IMTs) have been reported in various soft tissue and visceral anatomical locations. In 1994, the World Health Organization (WHO) defined IMT as an “intermediate soft tissue tumor that is composed of myofibroblasts-differentiated spindle cells accompanied by numerous inflammatory cells, plasma cells, and/or lymphocytes [4].” The most frequently reported site of IMT is the lung; however, it has occurred in the gastrointestinal tract, genitourinary tract, and the breast [5]. From the extrapulmonary cases, only 11% have been found in the upper respiratory tract, involving larynx, trachea, oropharynx, and nasopharynx. The other parts of the head and neck region resembling less than 5% of the cases involving the orbit, paranasal sinuses, major salivary glands, thyroid, and soft tissue of the face and neck in descending order of frequency [4–10]. The tumor occurs more frequently in children and young adults [11].

The etiology of IMT is largely unknown, with a few theories suggesting an exaggerated inflammatory response due to tissue injury of unknown origin. Other theories suggest that it could be due to a disruption in immunological responses [2]. Furthermore, some types of IMT are infection related. Epstein–Barr viruses have been linked to some hepatic and splenic IMTs, actinomyces, and mycoplasma in some pulmonary IMTs [1].

IMT is a benign lesion; however, it behaves as an aggressive one with extensive destruction [12]. However, several molecular and genetic reports suggest that some types of IMT are true monoclonal neoplasm [1]. In the short arm of chromosome 2, clonal rearrangements of anaplastic lymphoma tyrosine kinase (ALK) receptor have been reported in 50% of IMT cases involving the soft tissue [1]. ALK-1 expression is highly specific for IMTs, but it is not 100% sensitive [4].

IMT usually manifests as painless mass incidentally discovered over a short period of time, followed by the specific signs and symptoms depending on the site [10]. Intraorally, the lesion usually presents as a painless mass, which is firm and indurated upon examination [1]. In the nasal cavity and paranasal sinuses, the primary presentation is a gradually enlarging nonspecific sinonasal mass over a period of months or years [1]. Unlike IMTs involving the head and neck region, constitutional symptoms are often the chief complaints of IMT in visceral organs [1].

To the best of our knowledge, this case is the first reported case of an inflammatory myofibroblastic tumor to occur in the nasolabial fold. The patient underwent full radiological and pathological investigations to rule out any malignancy and to confirm the correct diagnosis.

IMT diagnosis is relatively nonspecific. On computed tomography scan and magnetic resonance imaging, the tumor usually manifests as an infiltrative growth, and it may mimic malignant characteristics due to possible bony changes including sclerosis, thickening, and erosions [1, 10]. CT scan of IMT with contrast usually demonstrate moderate enhancement with homogeneous tissue density [10]. On MRI, in relation to muscle, IMT commonly shows isointensity to hypointensity on T1-weighted images. In comparison to majority of tumors, IMT usually shows hypointesity on T2 signals [10]. Preoperative diagnosis is relatively difficult due to different clinical settings in which the tumor appears. Hence, histopathological examination is mandatory to reach an accurate diagnosis and avoid other invasive interventions for IMT [11]. Histopathological samples obtained by fine-needle aspiration biopsy or frozen section studies usually demonstrate a variable admixture of myofibroblasts and inflammatory cells, mainly lymphocytes, eosinophils, and plasma cells [10]. Three main histological patterns have been described, none of which have a clear association with the clinical presentation, namely, (1) myxoid/vascular pattern, resembling inflammatory granulation tissue; (2) compact spindle cell pattern with fascicular and/or storiform areas and variation of cellular density; and (3) hypocellular pattern, densely collagenised and reminiscent of a fibrous scar [1]. The three types may coexist all together within the tumor, with one or two types predominant. Myofibroblastic phenotype of the spindle cells can be confirmed by immunohistochemical studies, which are reactive to vimentin (99%), smooth muscle actin (92%), and muscle-specific actin (89%). Furthermore, the spindle cells may also be positive for desmin (69%) and cytokeratin (36%) [1, 10]. In this case, the nasolabial fold tumor cells were consistent with these histopathological and immunohistochemical features.

The management and prognosis of IMT is mostly favourable. The incidence of local recurrence of extrapulmonary IMT is 25% [1]. Thus, radical resection of the tumor is curative in 90% of extrapulmonary IMT, including IMT of the head and neck region [1]. The presence of ganglion-like cells, the combination of cellular atypia, p53 expression, and DNA aneuploidy can help to identify tumors with aggressive outcomes and high risk of recurrence [1].

The treatment of IMT by corticosteroid, radiotherapy, and chemotherapy has been beneficial either alone or in combination [10]. However, the gold standard management is total resection irrespective of the size due to destructive behaviour of the tumor [1]. In case surgery is contraindicated, radiotherapy is the best option [10]. Despite the relatively aggressive nature and rarity of IMT, it has a very good prognosis if treated properly.

## 4. Conclusion

IMT of the nasolabial fold is extremely rare. To our knowledge, this is the first reported case of IMT in the nasolabial fold. Due to its suspicious behaviour, histopathological examination and surgical excision are warranted. Radiological studies have limited value in diagnosis. Fortunately, if the tumor is completely resected, recurrence is extremely rare and prognosis is very good.
